# Genome-wide identification and expression analysis of *Wnt* gene family in the forest musk deer (*Moschus berezovskii*) under musk secretion stage

**DOI:** 10.1186/s12864-025-12451-7

**Published:** 2025-12-22

**Authors:** Yu-JiaWei Gu, Jun-Tao Sun, Fang Dan, Xue-Mei Jiang, Dan-Dan Liao, Cong-Xue Yao, Wen-Hua Qi

**Affiliations:** 1 College of Biological and Food Engineering, Chongqing Three Gorges University, Chongqing, 404100 P. R. China; 2 Chongqing Three Gorges Academy of Agricultural Sciences, Chongqing Three Gorges University, Chongqing, 404100 P. R. China; 3 Beijing Yanshe Biotechnology Development Co., Ltd., Beijing, 102200 P. R. China

**Keywords:** Forest musk deer, *Wnt*, Genome-wide identification, Expression analysis, Musk gland cell, RT-qPCR

## Abstract

The *Wnt* signaling pathway is ubiquitous in animals, playing a crucial role in embryonic development and adult tissue homeostasis in multicellular organisms. *Wnt* proteins act as ligands in this pathway, and their gene family encodes secreted signaling proteins involved in regulating vital physiological processes such as cell proliferation, migration, differentiation, polarity establishment, and maintenance. Based on the third-generation genome of the forest musk deer (FMD, *Moschus berezovskii*), this study aimed to identify members of the Wnt gene family, elucidate their physicochemical properties, analyze their chromosomal localization and gene structure, construct a systematic evolutionary tree, and predict the two-dimensional and three-dimensional structures of the Wnt protein family. Genome-wide identification was performed using BLASTP searches. Phylogenetic relationships were reconstructed using the Maximum Likelihood method, and 3D structures were predicted using SWISS-MODEL. Results revealed 18 *Wnt* (*MbWnt*) gene family members distributed across 8 chromosomes, with cDNA lengths ranging from 819 to 1900 base pairs in the FMD. The number of amino acids in the *Wnt* family proteins of the FMD ranged from 349 to 586aa, with isoelectric points (pI) between 7.96 and 10.24, and molecular weights concentrated between 30 and 62 kilodaltons. Signal peptide analysis showed that only *MbWnt1*, *MbWnt2*,* MbWnt4*,* MbWnt6*, and *MbWnt9b* contained signal peptides. Subcellular localization analysis indicated that the Wnt gene family is primarily located in the nucleus and cytoplasm. *MbWnt* proteins shared six conserved motifs. Phylogenetic analysis demonstrated high consistency in *Wnt* conservation and grouping in the genomes of cattle (*Bos taurus*), goat (*Capra hircus*), sheep *(Ovis aries)*, and red deer (*Cervus elaphus*). Genome collinearity analysis demonstrated substantial chromosomal correspondence and orthologous conservation between the FMD and the three species of Bovinae and Ce. elaphus. The level of chromosome homology is relatively low, but the level of genome homology is relatively high among these species. Musk gland transcriptomic datasets indicated that the expression of *MbWnt* genes were stage-specific at musk secretion stage and non-secretory stage. RT-qPCR analysis indicated that six *MbWnt* genes were significantly highly expressed during the musk secretion stage, whereas two genes showed no significant differential expression.

**Supplementary Information:**

The online version contains supplementary material available at 10.1186/s12864-025-12451-7.

## Introduction

The Wnt gene family is an important class of secreted glycoproteins [1], which typically consist of 350–400 amino acids and are characterized by 23–24 conserved cysteine residues [2–4]. Wnt genes are found in diverse species ranging from cnidarians to mammals. While these Wnt genes show less similarity between vertebrates and invertebrates, they are still considered a conserved gene family throughout evolution [2]. Wnt proteins typically contain four common domains: (1) signal peptide that facilitate secretion; (2) a lipid modification site involved in lipid modification at the C-terminus, anchoring the protein to the cell membrane; (3) a Wnt binding domain that interacts with receptor proteins to mediate signaling; (4) a cysteine-rich domain with multiple cysteine residues essential for protein formation and function. The spatial arrangement and interactions of these domains determine the structure and function of Wnt proteins. The Wnt signaling pathway exhibits ubiquitous distribution across both invertebrate and vertebrates, which is highly conserved passagegeway in species evolution. The main mechanisms of Wnt signaling are as follows: the canonical Wnt/β-catenin, non-canonical Wnt/Ca^2+^, and non-canonical planar cell polarity (PCP). Each of these signaling modalities necessitates specific binding interactions with the transmembrane receptor Frizzled (FZD) to orchestrate appropriate intracellular responses. In the canonical Wnt pathway, Activation of the Fzd receptor promotes the stabilization and cytoplasmic accumulation of β-catenin, which subsequently translocates into the nucleus [5]. Within the nuclear compartment, β-catenin binds to the transcription factor T-cell factor/lymphoid enhancer factor (LEF/TCF) family. This molecular partnership initiates the transcriptional activation of target gene governing embryonic morphogenesis, tissue regeneration processes, and cellular proliferation and differentiation programs [5, 6]. The formation of this regulatory complex drives the transcription of downstream target genes that orchestrate essential biological processes, including embryonic development, tissue regeneration, as well as cellular proliferation and differentiation [5, 6].

The number of Wnt genes varies among different species. Thirteen Wnt gene subfamilies have been identified, including *Wnt1* to *Wnt11*, *Wnt16*, and *WntA* [7]. The chicken Wnt gene family has 39 members, the silkworm *Bombyx mori* 8, Cattle *Bos taurus* 19, mouse *Mus musculus* 19, catfish Silurus asotus 20, The African clawed frog *Xenopus laevis* 20, and human *Homo sapiens* 19 [8, 9]. Notably, the *WntA* subfamily has been lost in vertebrates. The molecular function of Wnt proteins is highly conserved, which play a significant role in growth and development, participating in various physiological processes and maintaining homeostasis [10, 11]. Members of the Wnt gene family exhibit functional differentiation: *Wnt1* is involved in nervous system development and tumorigenesis; *Wnt3* regulates embryonic development and stem cell self-renewal; *Wnt5* affects cell migration and polarity; *Wnt6* is involved in organ development and cell proliferation; *Wnt7* plays a role in the development of the nervous system and muscles; *Wnt8* is involved in embryonic development and cell polarity regulation. In the atypical Wnt/PCP pathway, *Wnt5a* and *Wnt11* regulate cytoskeletal actin and cell polarization. Ectopic expression of *Wnt1* stabilizes β-catenin and activates TCF-dependent gene transcription, thereby blocking adipogenesis. Based on their mechanisms in development and disease, Wnt proteins can be divided into those promoting stem cell proliferation and those participating in nervous system development. The typical Wnt signaling pathway is crucial in maintaining undifferentiated precursor adipocytes by inhibiting adipogenesis. It has been reported that the Wnt gene participated in the development process of various glands (such as uterine glands, alveoli, etc.) by regulating stem cell differentiation and activating signaling pathways [12]. At present, the role of Wnt genes in the musk gland of the forest musk deer (FMD, *Moschus berezovskii*) remains unknown.

FMD is a ruminant animal belonging to the musk family and the musk genus, and is also an important economic animal. Male musk deer have musk glands that can secrete musk [13–15]. Musk is not only a kind of traditional Chinese medicine, but also an important raw material of advanced perfume and a fixative of essence. As a traditional Chinese medicine, musk has an exciting effect on stimulating the central nervous system and can be used to prepare emergency drugs such as resuscitation and heart strengthening, which has high economic and medicinal value [16]. The Wnt gene has been systematically identified in species such as cattle, mice, chickens, fish, and silkworms, and has been shown to play an important regulatory role. However, there have been no reports on the Wnt gene family of the FMD entire genome [14–17]. With the completion of the third-generation genome sequencing of the FMD, there is an opportunity to explore the biological significance of functional genes in the FMD. Systematic identification of Wnt family members in the FMD entire genome can help reveal their gene structure and function, and explore their regulatory mechanisms. This study used multiple biological methods to identify the Wnt gene family of the FMD chromosomal-level genome, and characterized its physicochemical properties, gene structure, conserved domains, collinearity, advanced structure, and systematic evolution. Transcriptome data is used to analyze the expression profile of Wnt genes in musk gland tissues at different stages, laying the foundation for further elucidating the potential function of the Wnt protein family in gland cell differentiation. And the expression profiles of these Wnt genes in the normal group and stress group of cultured musk gland cells are also analyzed in the FMD by using RT-qPCR.

## Materials and methods

### Genome-wide identifcation of *Wnt* genes

Genomic sequences and their corresponding annotations for cattle (*B. taurus*, ENSBTAG00000074665.1), sheep (*O. aries*, ENSOARG00020093025.1), goat (*Ca. hirus*, ENSCHIG00000008572.1), and red deer (*Ce. hanglus*, ENSCHYG00000000002.1) were obtained from the Ensembl database(https://www.ensembl.org). The chromosome-level genome and annotation of FMD were retrieved from MuskDB (http://muskdb.cn/home). For the comprehensive identification of putative *Wnt* gene homologs within the Moschidae family, we employed the Basic Local Alignment Search Tool (BLAST)( https://blast.ncbi.nlm.nih.gov/Blast.cgi). A total of *Wnt* sequences of *B. taurus* (7), *Ca. hirus* (7), *O. aries* (13), *Ce. elaphus* (19), and *H. sapiens* (19) all retrieved from the UniProt database and used to detect candidate *Wnt* genes via BLASTP with a threshold e-value of 10^–5^.

### Phylogenetic tree construction,chromosomaldistribution,and collinearity analysis

Protein sequences were aligned using ClustalW (https://www.genome.jp/tools-bin/clustalw) to investigate the phylogenetic relationship of *Wnt* genes. A Maximum likelihood tree was constructed in MEGA using the bootstrap method (Boostrap number: 5000), the Poisson model, and complete deletion. ITOL (https://itol.embl.de/) was used to adjust and enhance the evolutionary tree. The chromosomal locations of *Wnt* genes in the FMD were obtained from general feature format (GFF3) files. Gene Location Visualize from GFF was used to map the distribution of *Wnt* genes. Collinearity analysis for orthologous genes between FMD and the four other species was performed using the MCScanX toolkit. Subsequently, genome collinearity results and orthologous *Wnt* genes were visualized by Dual Systeny Plot for MCscanX.

### Analysis of *Wnt* protein characteristics

The physicochemical characteristics of proteins encoded by the identified *Wnt* genes were analyzed using the ExPASy ProtParam tool(https://www.expasy.org/). Conserved sequence motifs were predicted with MEME 5.050, specifying minimum and maximum motif lengths of 351 and 586 amino acids, respecively. The exon–intron structures and motif patterns of the *Wnt* family were visualized using TBtools (v2.133) software. NCBI Conserved Domain Database (CDD; https://www.ncbi.nlm.nih.gov/Structure/cdd/cdd.shtml) was used to identify the conservative domains. The conserved domains were identified by using the NCBI. Multiple sequence alignments were generated for *Wnt* proteins, and a phylogenetic Maximum likelihood tree were built by using the MEGA 6.0 sofware.

### Conservative motif and gene structure analysis of *Wnt* family members in the FMD

The MEME (Multiple Expectation Maximization for Motif Elicitation, https://meme-suite.org/meme/tools/meme) database was used to analyze the conserved motifs of *Wnt* protein members in FMD, which were visualized by using TBtools software. TBtools was also employed to analyze and visualize the structure of *Wnt* gene family members in FMD.

### Chromosome localization and collinearity analysis of *Wnt* gene family in FMD

Extract the chromosomal location information of *Wnt* gene family members from the annotated genome files of FMD, and use TBtools software to map these genes onto the corresponding chromosomes. TBtools was also used to determine the collinearity relationships of the *Wnt* family among FMD, cattle, goats, and sheep, and to visualize these relationships between species.

### Transcriptome and expression analyses of *MbWnt* genes in the musk gland tissues at different stages

The gene expression data of FMD musk gland tissues at different stages were downloaded from MuskDB [18]. The raw reads has undergone quality control. The index of the reference genome were constructed by using Hisat2, and the clean reads were aligned with the reference genome [19]. Then, FPKM (fragments per kilobase million) value for each gene was computed to represent the expression level of gene.

### Enrichment analysis of *Wnt* gene family in the FMD

Enrichment analysis and visualization of the selected 18 *MbWnt* genes were conducted by using the Gene Ontology (GO) and Kyoto Encyclopedia of Genes and Genomes (KEGG) databases.

### Analysis of *Wnt* gene family interaction network in musk deer

Based on these 18 members, the STRING database was used to study the interaction network of homologous proteins. The network was optimized and visually analyzed using Cytoscape software.

### RNA extraction and qRT-PCR

Total RNA extraction from cellular samples was accomplished by utilizing the GOONIE Fast RNA Extraction Kit protocol. RNA concentration quantification was performed via microspectrophotometric analysis using a HIPIE instrument (Hangzhou, China). Complementary DNA (cDNA) synthesis was conducted employing the Fast First-Strand cDNA Synthesis Mix for RT incorporating dsDNase treatment. Oligonucleotide primer sequences were computationally designed through the Primer 3 (Table 1), with β-actin (ACTB) designated as the internal reference gene. The RT-qPCR using a standardized 20 µL reaction volume, which contain 0.4 µL primer solutions (both forward and reverse primer), 2 µL template cDNA (1000 ng/µL concentration), 10 µL qPCR SYBR Green Fast Taq master mix, and 7.2 µL DEPC water. Thermal cycling parameters comprised initial denaturation at 95 °C for 30 s, succeeded by 40 amplification cycles consisting of denaturation at 95 °C for 10 s and primer annealing/extension at 60 °C for 30 s. Melting curve analysis was subsequently performed through temperature transitions from 95 °C (15 s) to 60 °C (60 s) to verify amplicon specificity. All experimental samples were subjected to technical triplication.

**Table 1:** The primer sequences of *MbWnts*

Primer Name	Sequence(5′-3′)
*MbWnt1*	F: CGACAACATCGACTTCGGTC
	R: CGCATCTCGGAGAAAACGGT
*MbWnt1s*	F: CCTGCTCGCCTTGCTGTTC
	R: TGATGCTGGCACTCCCTGAT
*MbWnt2*	F: TGACTGAGTGGACGATGGA
	R: CTGGTGATGGCAAATACAA
*MbWnt4*	F: GTCGTCCGTGGGCAGCATCT
	R: GAGTCGAGCGTGGAGCAGTT
*MbWnt5a*	F: GGCTGGAAGGGCAATGTCT
	R: CGGTGTCGGAACTGATACTGG
*MbWnt7a*	F: GGAGAAGGCTCGCAAATGG
	R: CGATGCCGTAGCGGATGT
*MbWnt7b*	F: CCAACTACTGCGAGGAGGACG
	R: GCACTTGACGAAGCAGCACC
*MbWnt16*	F: TTCGGCTTGTATCAGTATTCC
	R: GTCGCATTCCAAGGTGAG
*ACTB*	F: CACCGCAAATGCTTCTAGGC
	R: TGTCACCTTCACCGTTCCAG

### Acquisition and culture of cell samples

musk gland cells were obtained from Beijing Yanshe Biotechnology Development Co., Ltd., The cells were cultured in meilunbio’s DMEM/F-12(Dulbecco’s Modified Eagle Medium/Nutrient Mixture F-12) supplemented with L-Glutamine(365 mg/L) and Sodium Pyruvate (55 mg/L) and Phenol Red(8.1 mg/L). All cells were incubated at 37 °C in a humidified atmosphere containing 5% CO2.

### Statistical analysis

All RT-qPCR results were determined using the 2 − ΔΔCt method. 3 independent technical repetitions were performed for each test.The raw Ct values used for calculation are provided in Supplementary Table S3.Statistical significance was determined using GraphPad Prism 7.0 software.

## Results

### The *Wnt* gene repertoire in the FMD

**Fig. 1:**
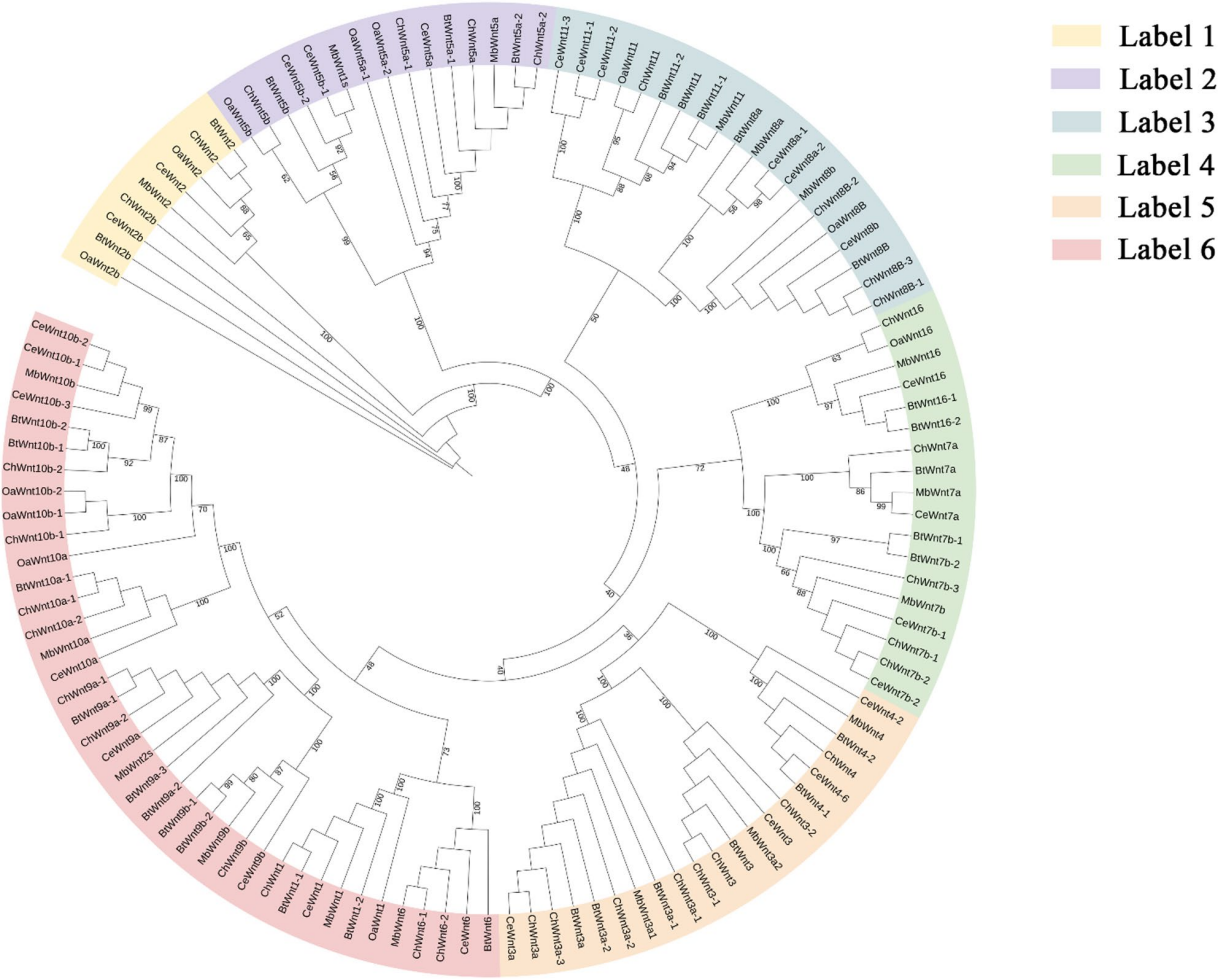
Phylogenetic relationships of *Wnt* proteins in *B. taurus*, *O. aries*, *Ca. hircus*, *Ce. elaphus* and FMD that were established by the Maximum Likelihood (ML) connection method within the TBtools software

A comprehensive dataset comprising 65 *Wnt* amino acid sequences served as reference queries for the systematic identification of *Wnt* family members. These validated sequences were sourced from cattle (*B. taurus*, 7), goat (*Ca. hirus*, 7) sheep (*O. aries*, 13), red deer (*Ce. elaphus*, 19), and human (*H. sapiens*, 19). Based on the genome-wide detection of homologous sequences, 18 *Wnt* genes were successfully identified in the FMD genome (Fig. 1a ). In the FMD, the amino acid sequences of the 18 *Wnt* proteins ranged from 349 residues (*MbWnt7a*) to 586 residues (*MbWnt11*); with corresponding molecular weights from 38972.82 to 64827.77 Da. Isoelectric point (pI) analysis demonstrated that all proteins, with the singular exception of *MbWnt3a1* (pI = 7.96), the rest of the *Wnt* proteins had a pI higher than 8.0, consistent with their high content in basic amino acids. All 18 *Wnt* proteins possessed the *Wnt* conserved domain (Fig. 1a). Overall, the isoelectric points (pI) of these proteins range from 7.96 to 10.24, indicating that they are all alkaline proteins among the members of the *Wnt* protein family in the FMD, except for *Wnt5a* and *Wnt7a*, which are considered unstable, most of them are stable proteins. These protein hydrophobicity indices range from − 0.533 to -0.271, all of which are negative values, indicating that members of the *Wnt* gene family in the FMD are hydrophilic proteins (Table 2).

**Table 2:** Protein haracteristics analysis of Wnt family genes in the FMD

Gene name	Amino acid number	pI	Molecularweight/D	Instability index	Gravy	Subcellular Localization	Signal peptide
*MbWnt1*	370	9.28	40992.65	61.14	-0.364	extr, nucl	YES
*MbWnt1s*	358	8.94	40041.93	42.58	-0.347	extr, lyso	YES
*MbWnt2*	360	9.25	40596.64	43.13	-0.404	extr, E.R., golg:	YES
*MbWnt2s*	366	9.12	40496.48	49.83	-0.358	extr, lyso	YES
*MbWnt3a1*	352	7.96	39400.6	47.86	-0.388	plas, nucl, cyto_nucl:, extr, cyto_pero, golg	NO
*MbWnt3a2*	579	8.67	63567.1	48.48	-0.444	plas, nucl:, cyto_nucl, cyto, mito, golg	NO
*MbWnt4*	351	8.92	39118.82	51.82	-0.271	extr, mito, lyso, cyto	YES
*MbWnt5a*	380	8.88	42408.56	38.37	-0.316	extr, plas, nucl, lyso	NO
*MbWnt6*	365	9.15	39706.77	47.78	-0.283	extr, plas, E.R., lyso	YES
*MbWnt7a*	349	9.05	38972.82	35.8	-0.393	mito, cyto_mito, extr, cyto, plas	NO
*MbWnt7b*	472	9.86	52399.24	55.82	-0.483	cyto_nucl, nucl, cyto, mito, plas, E.R.	NO
*MbWnt8a*	351	8.45	39199.51	52.14	-0.377	extr, lyso, plas, cyto, mito	NO
*MbWnt8b*	379	8.91	41972.45	52.62	-0.495	nucl, cyto_nucl, cyto	NO
*MbWnt9b*	448	8.85	48886.58	53.53	-0.460	extr, E.R., plas, lyso	YES
*MbWnt10a*	417	9.36	46341.19	63.14	-0.437	plas, extr, E.R., lyso	YES
*MbWnt10b*	391	9.42	43020.34	50.66	-0.406	extr, plas, nucl, cyto, lyso	YES
*MbWnt11*	586	10.24	64827.77	51.55	-0.533	plas, nucl, extr_plas, cyto_nucl, cyto, extr:, mito	NO

*Nucl* Nucleus, *Cyto* Cytosol, *Chlo* Chloroplast, *Extr* Extracellular, *Cysk* ytoskeleton, *Vacu* Vac-uole, *Mito* Mitochondria, *Plas* Plasma membrane, *GRAVY* Grand average of hydropathicity, *Golg* Golgi apparatus, *Pero* Peroxisome, *E.R* Endopplasmic reticulum

### Phylogenetic relationship of *Wnt* proteins in different species

Phylogenetic reconstruction serves as a fundamental framework for elucidating the evolutionary trajectory and functional diversification patterns of the *Wnt* gene family. We also included the *Wnt* proteins from well-studied model organisms (*B. taurus*, *O. aries*, *Ca. hircus*, and *Ce. elaphus*). Of these five species, 122 amino acid sequences were aligned to generate a Maximum Likelihood (ML) tree (Fig. 1) within the TBtools software, comparative sequence analysis revealed that *Wnt* proteins from *B. taurus*, *O. aries*, *Ca. hircus*, and *Ce. elaphus* exhibited high sequence homology with their counterparts in the reference genome, The *Wnt2*,* Wnt3*,* Wnt5*,* Wnt7*,* Wnt8*,* Wnt9*, and *Wnt10* subfamilies had two members. whereas the *Wnt1*,*Wnt4*,*Wnt6*, and *Wnt16* subfamilies had one members.

### Structural features of *Wnt* family members in the FMD

**Fig. 2:**
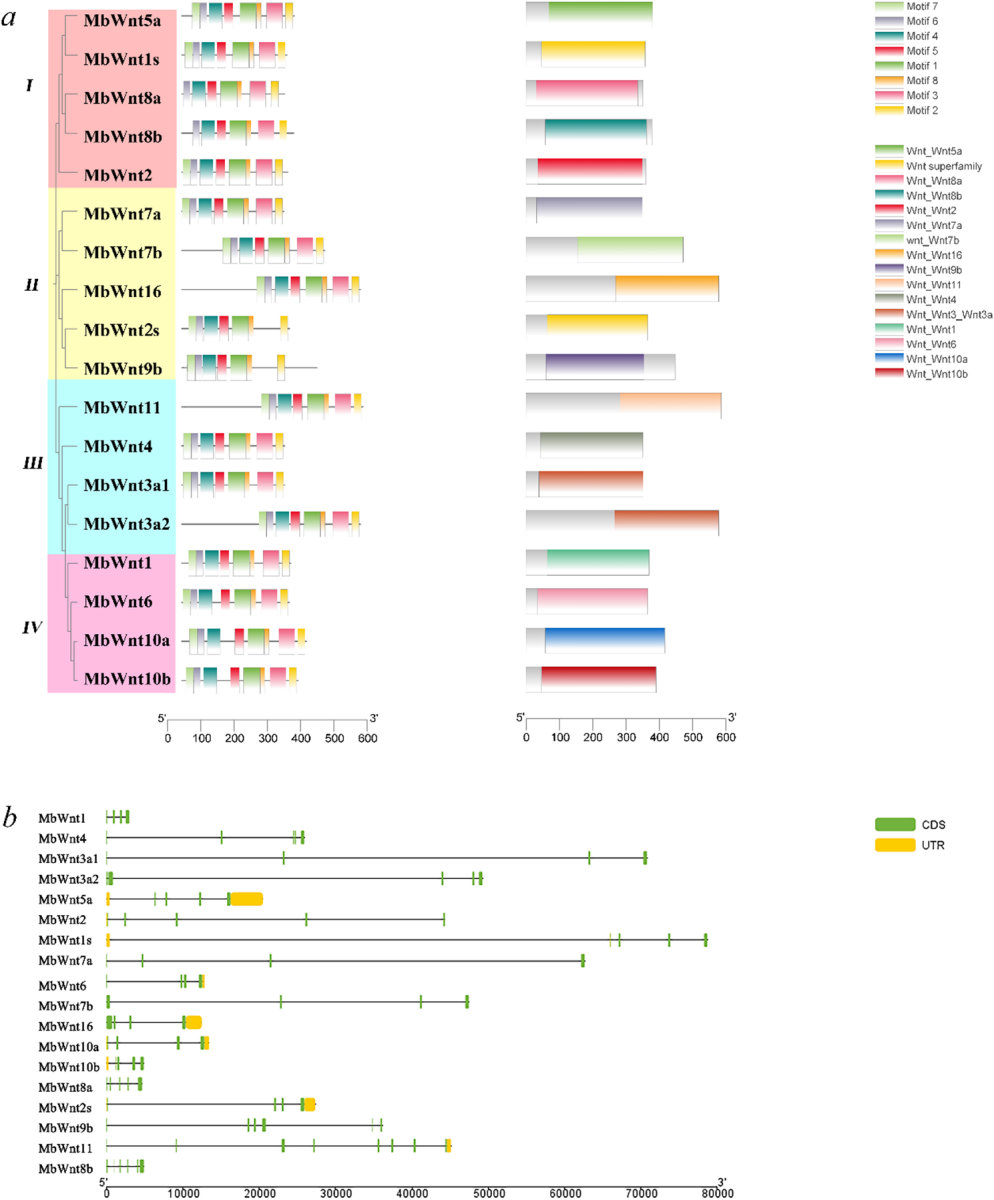
**a **Conservative motifs analysis of *MbWnts* and genetic structure diagram of *MbWnts* (**b**) Schematic diagrams of the domains of the *MbWnts* gene family. Exons and 5′ UTR/3′ UTR are displayed using yellow bars and green bars. Black lines denote introns

The phylogenetic relationships were predicted based on the conserved motifs and gene structures (Figs. 2a).The 18 *MbWnt* genes clustered into four distinct phylogenetic subfamilies (I–VI). All *MbWnt* proteins shared six conserved domains, namely motifs 1, 2, 4, 5, 6, and 8 formed by 50, 49, 41, 29, 28, and 9 amino acids, respectively. Only the *MbWnt8a* and *MbWnt8b* demonstrated the absence of motif 7. while *MbWnt2* and *MbWnt9b* lacked motif 3. The remaining 14 family members contain all 8 motifs. The distributions of introns, coding sequences (CDS), and untranslated regions (UTR) were variable among the *MbWnt* gene family (Fig. 2b). Despite this variability, the *MbWnt* gene members in the same subfamily often have similar gene structure patterns and conserved motifs (Fig. 2a).

### Prediction of protein structure of *Wnt* gene family in the FMD

**Fig. 3:**
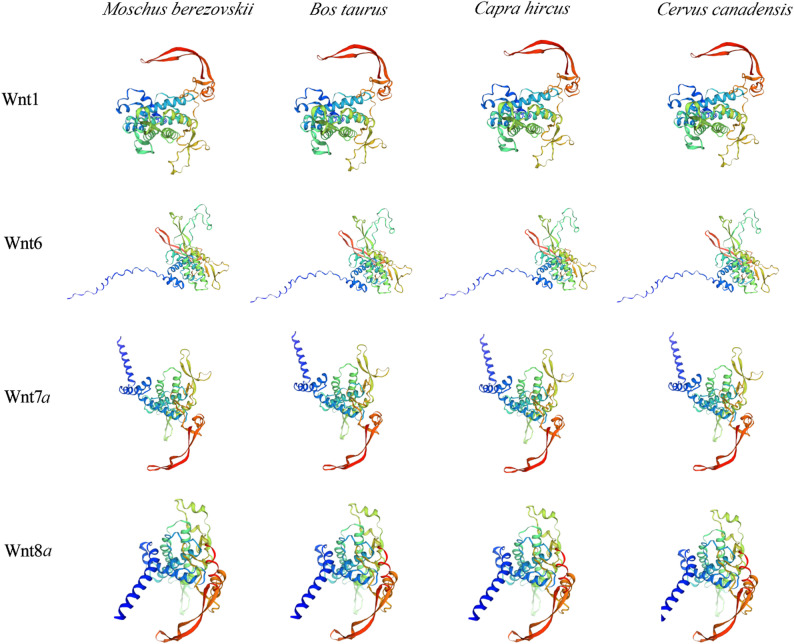
Three-dimensional structure of *Wnt* family proteins of M.berezovskii, B. taurus, Ca. hircus and Ce. elaphus

The predicted protein structure of *MbWnt* gene family was shown in (Supplementary Table S1). Irregular curling is the main structure of the *MbWnt* family proteins, accounting for an average of 47%. Especially, the *MbWnt3a2* protein has the highest proportion of irregular curls (55.96%), next are alpha helices and beta angles, accounting for 26.08% and 4.49%, respectively. A three-dimensional structural model of *Wnt* protein was successfully constructed through homology modeling. Comparing the protein three-dimensional structure of the *Wnt* gene family in the FMD with that of *Wnt* proteins in cattle, goats, and sheep (Fig. 3), it was found that these proteins generally contain larger AlphaFold regions and exist in monomeric form. The results indicate that *Wnt1*,* Wnt6*,* Wnt7*,* and Wnt8* proteins have similar structures in these species. Overall, proteins from different species within the same subfamily exhibit similar structures, while proteins from different subfamilies within the same species show significant differences. This result reveals the structural diversity of the *Wnt* gene family.

### Chromosomal distribution and collinearity analysis of *Wnt* genes

**Fig. 4:**
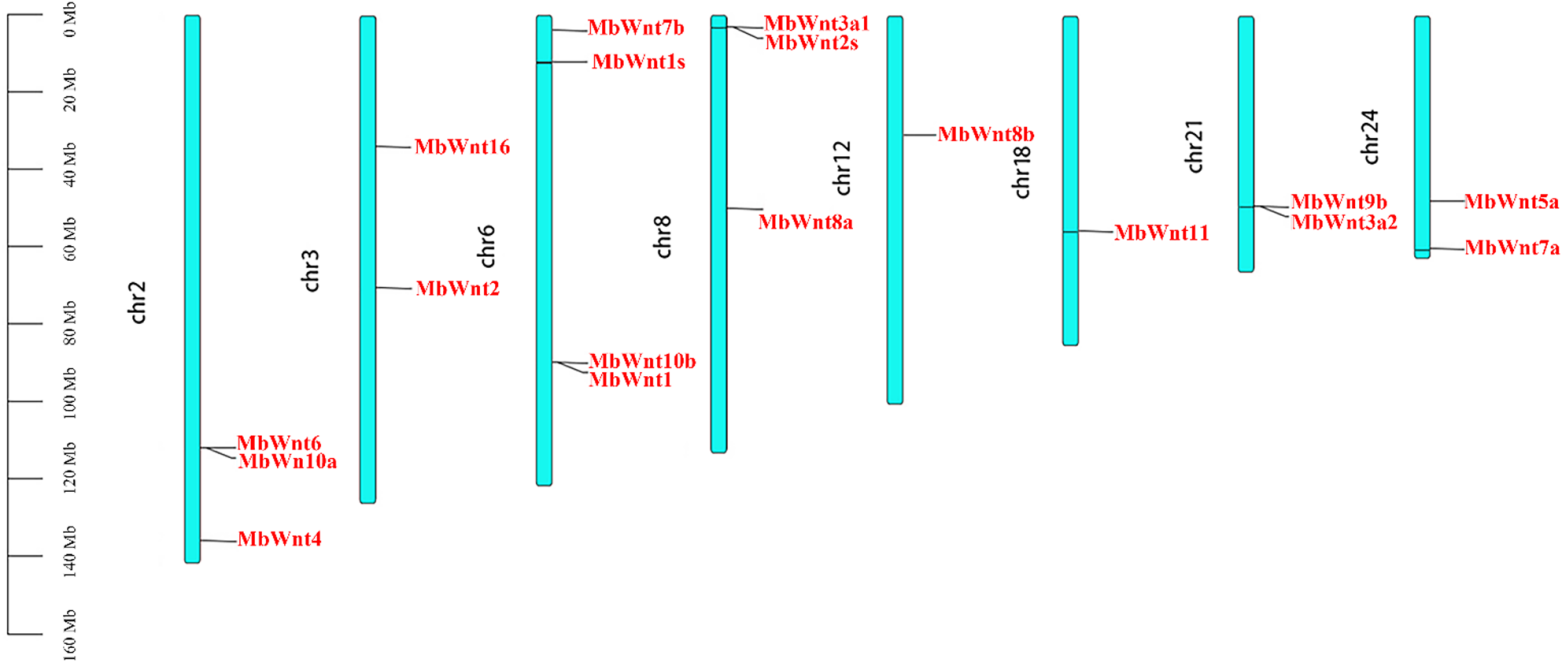
Chromosome location diagram of the *MbWnts*

Members of the *MbWnt* genes are only partially distributed on the chromosomes and are scattered, with 18 *MbWnt* genes distributed across eight chromosomes, most of them are far from the telomere area. Only *MbWnt7a*,* MbWnt7b*,* MbWnt2s*, and *MbWnt3a1* are located in the telomere region (Fig. 4).

**Fig. 5:**
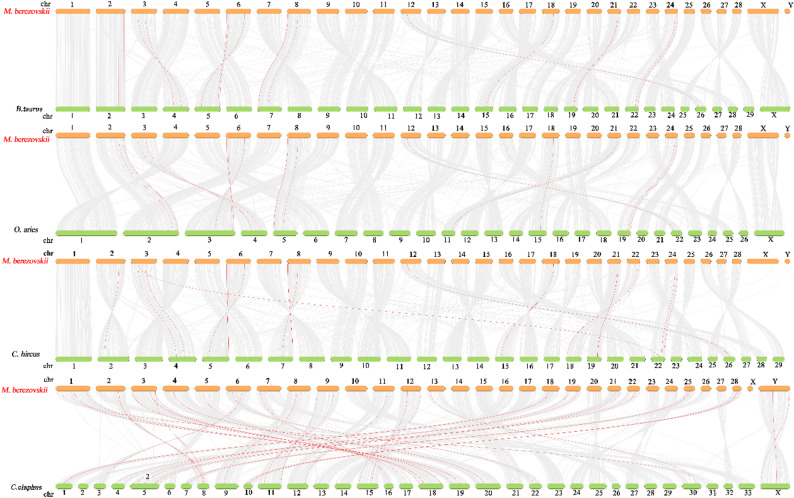
Interspecific collinearity relationship of *Wnt* genes between *M.berezovskii*, *B. taurus*, *O. aries*, *Ca. hircus* and *Ce. elaphus*

Genome collinearity analysis demonstrated substantial chromosomal correspondence and orthologous conservation between the FMD and *B. taurus*, *O. aries*, *Ca. hircus*, and *Ce. elaphus* (Fig. 5). FMD had 17 collinearities with *B. taurus*, 15 collinearities with *O. aries*, and 17 collinearities with *Ca. hircus*, and 17 collinearities with *Ce. elaphus*. The level of chromosome homology is relatively low, but the level of genome homology is relatively high among these species. In addition, collinearity modules explained the difference in the position of the *Wnt* gene family in FMD relative to the other three species in Bovinae and the one species in Cervidae. Most of the *Wnt* genes are distributed on different chromosomes between FMD and other four species, which may be caused by inter-chromosomal rupture or fusion during the evolution. *Wnt4* and *Wnt10a* are distributed on same chromosomes (Chr 2) between FMD and the bovid species, but the position variation of *Wnt4* and *Wnt10a* between FMD and *Ca. hircus* might have been caused by intra-chromosomal translocation events (Fig. 5).

### KEGG and GO enrichment analysis of *Wnt* gene family in the FMD

**Fig. 6:**
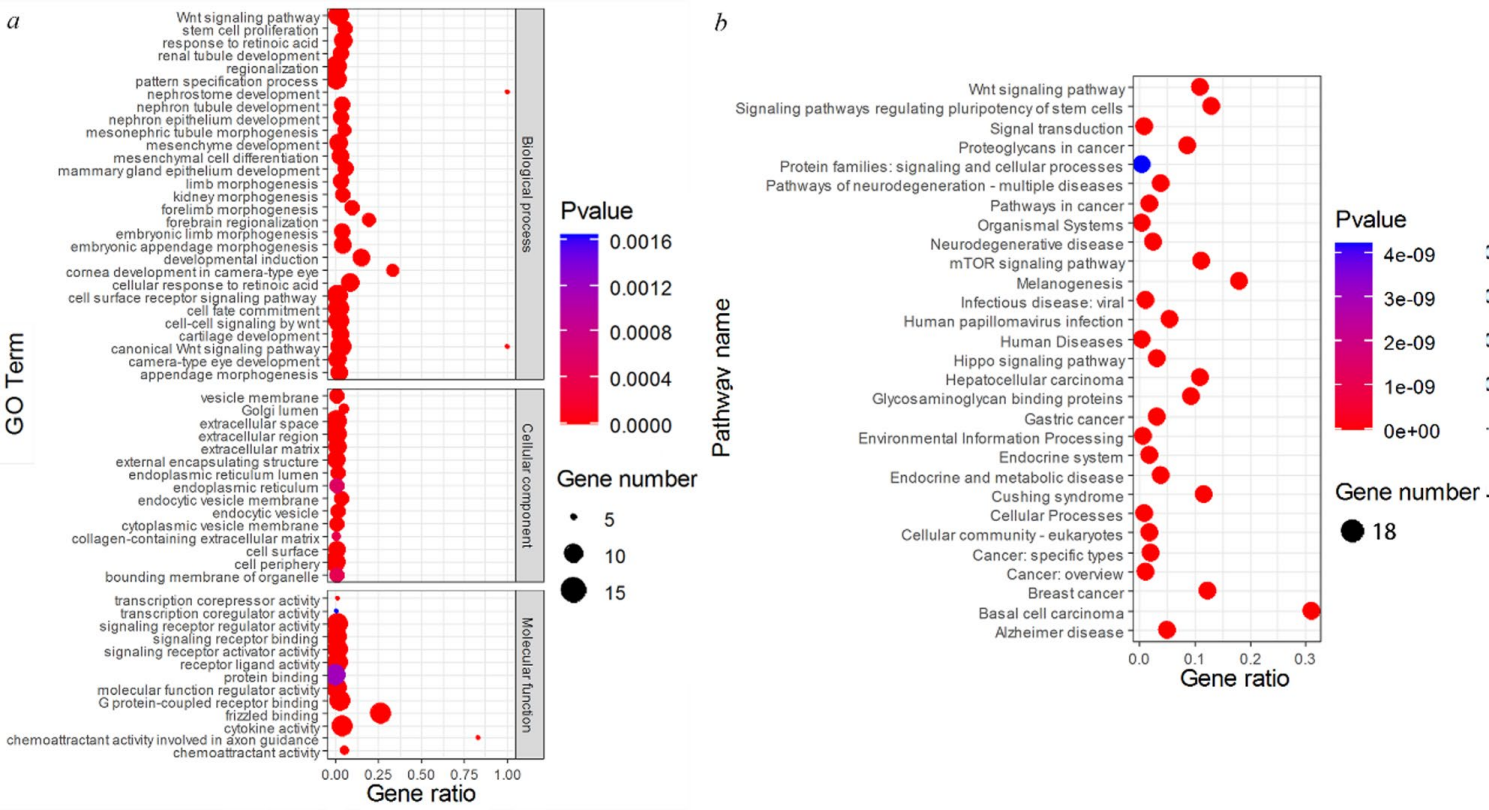
**a** GO enrichment analysis of *Wnt* gene family in the FMD. **b** KEGG enrichment analysis of *Wnt* gene family in the FMD

In order to explore the biological functions of 18 *Wnt* genes in the FMD, GO and KEGG analysis were performed. 18 members of the *Wnt* gene family in the FMD were annotated into 603 categories (GO Terms), including 15 molecular functions, 24 cellular components, and 564 biological processes (*p* < 0.05; Supplementary Table S2). In the BP category, the *MbWnt* genes were obviously enriched in the embryonic limb morphogenesis, cartilage development, forelimb morphogenesis, nephron epithelium development, nephron tubule development, stem cell proliferation, developmental induction, *Wnt* signaling pathway, and so on (Fig. 6a; Supplementary Table S2). Multiple terms of these BPs have been reported to be connected with Cell proliferation, embryogenesis and organ development.

**Fig. 7:**
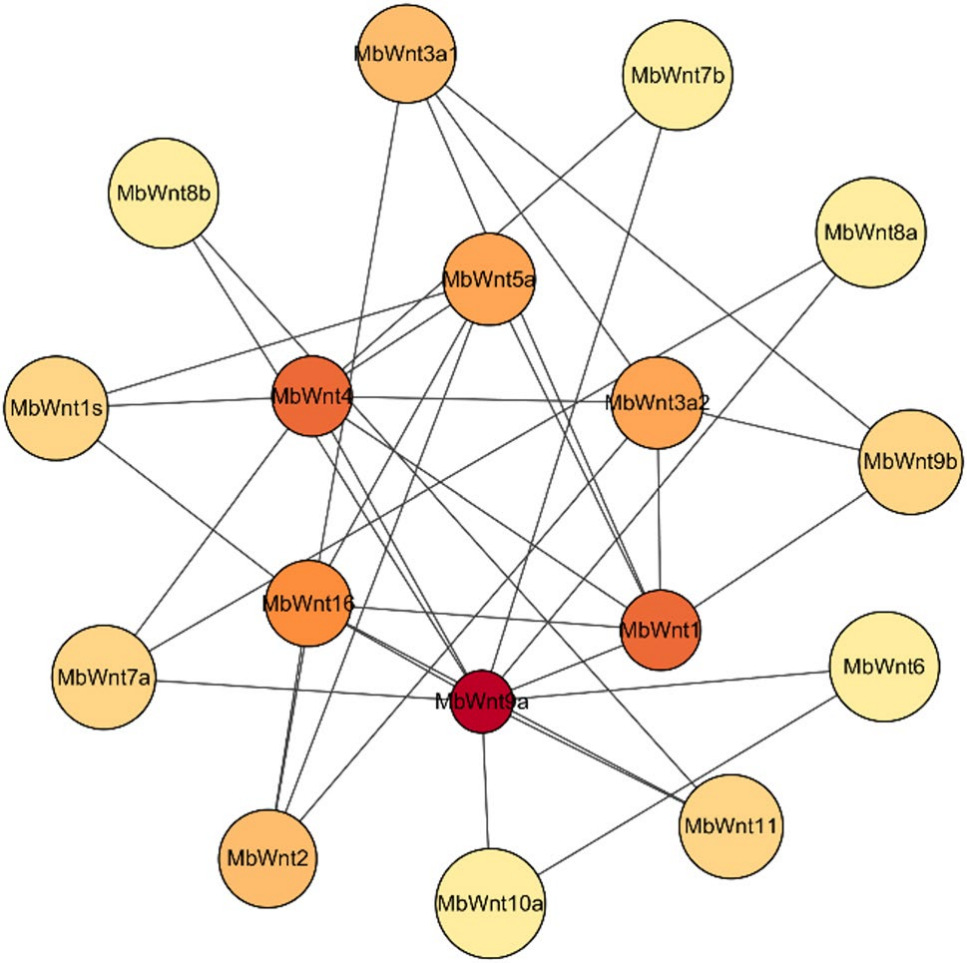
Analysis of *Wnt* gene family interaction network in the FMD

29 significant pathways were found by the KEGG analysis of 18 *MbWnt* genes (Fig. 6b; Supplementary Table S2). These significant pathways were recognized as follows: *Wnt* signaling pathway, Glycosaminoglycan binding proteins, Pathways of neurodegeneration in multiple diseases, Gastric cancer, Hippo signaling pathway, Proteoglycans in cancer, Alzheimer disease, Signaling pathways regulating pluripotency of stem cells, and so on (Fig. 7b; Supplementary Table S2). Most of these pathways were involved in signaling pathways and multiple diseases in previous studies.

### Analysis of *Wnt* gene family interaction network in the FMD

In our analysis, we projected a total of 35 gene-gene pairs in the FMD. The hub genes identified in one of the MCODE models in the PPI network including *MbWnt9a*,* MbWnt1*,* MbWnt4*,* MbWnt16*, *MbWnt3*,* MbWnt5a*,* MbWnt3a*,* MbWnt2*,* MbWn5b*, and *MbWn5b* major belonged to Wnt signaling pathway (Fig. 7). It can be seen from the figure that the interaction intensity between the *MbWnt9a* and the *MbWnt1*,* MbWnt16*,* MbWnt4*,* MbWnt6*, and *MbWnt10a* are relatively high. These proteins mainly participate in the regulation of embryonic development and cell proliferation. And the interaction intensity between the *MbWnt1* and the *MbWnt4*,* MbWnt3a*,* MbWnt5a* and *MbWnt9b* are relatively high. These proteins are mainly involved in developmental regulation, disease occurrence and cell proliferation and differentiation.

### Expression profiling of *MbWnt* genes in the musk gland tissues at different stage

**Fig. 8:**
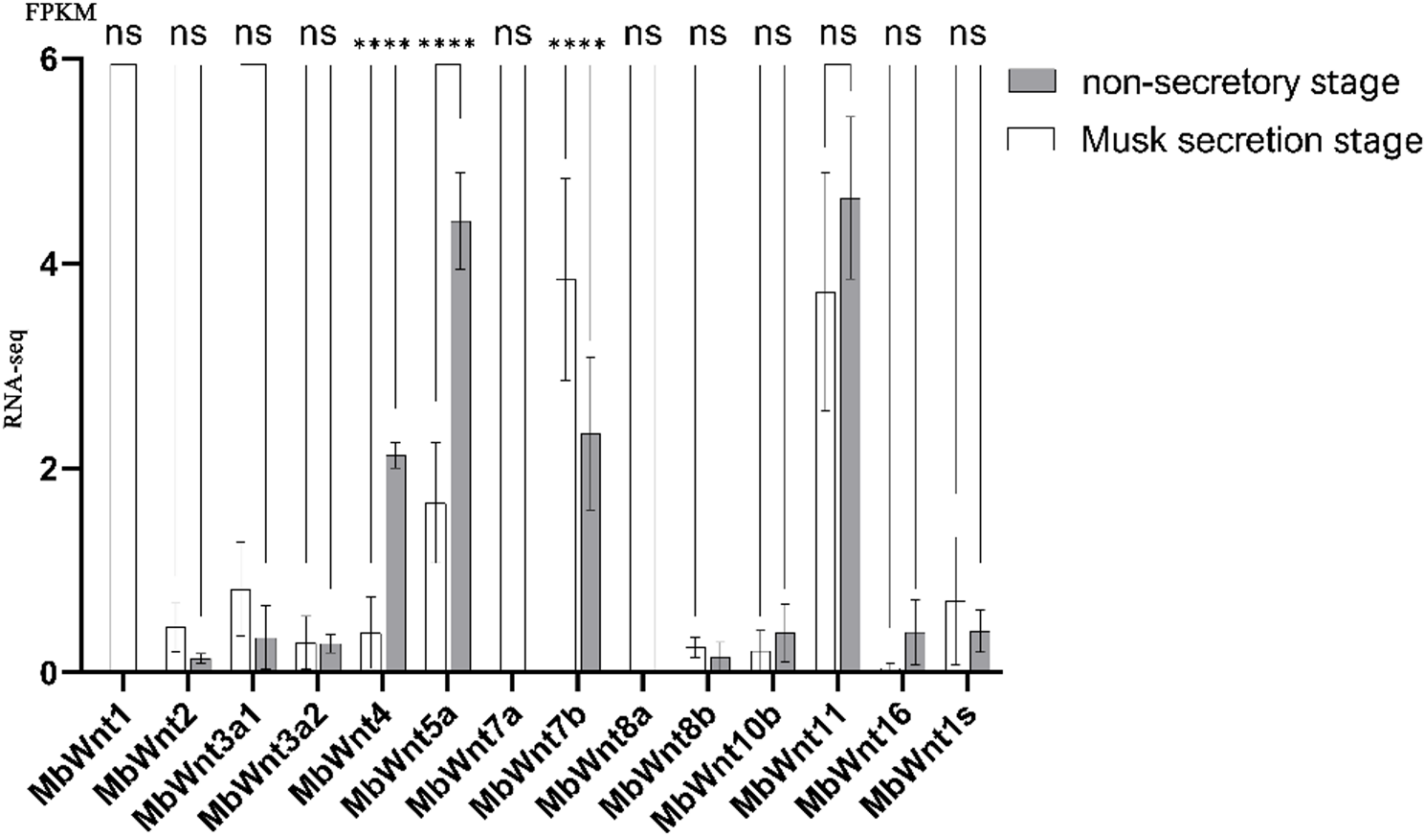
Expression profiling of *MbWnt* genes in the musk gland tissues at different stage

In order to investigate the specific expression of *MbWnt* gene, we analyzed the RNA-seq data of *MbWnt* genes at two developmental stages, including non-secretory stage and musk secretion stage. As is shown in Fig. 8, two *MbWnt* genes (*MbWnt4*,* MbWnt5a*) were significantly down-regulated and one *MbWnt* gene (*MbWnt7b*) was significantly up-regulated in the musk secretion stage, whereas two *MbWnt* genes (*MbWnt4*,* MbWnt5a*) were significantly up-regulated and one *MbWnt* gene (*MbWnt7b*) was significantly down-regulated in the non-secretory stage. In contrast, the *MbWnt7b* gene showed a stage-specific significantly high expression in the musk secretion stage, the *MbWnt4*, and *MbWnt5a* gene showed a stage-specific significantly high expression in the non-secretory stage.

### Validation of *MbWnt* genes in vitro culture of musk gland cell by RT-qPCR

**Fig. 9:**
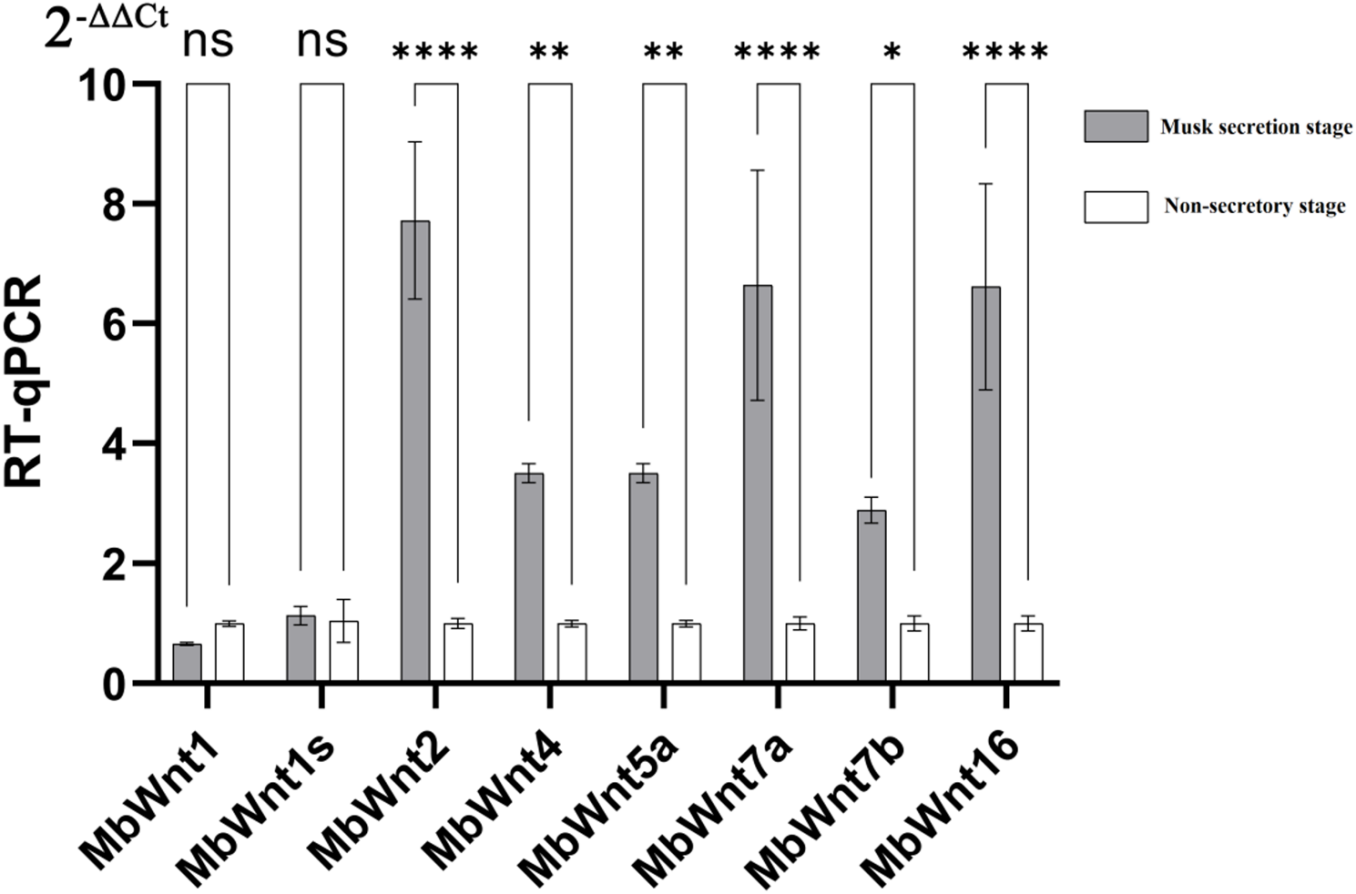
Validation of *MbWnt* genes in vitro culture of musk gland cell by RT-qPCR

To verify the specific expression of the *MbWnt* genes, we selected the cultured musk gland cell at two developmental stages, including non-secretory stage (C) and musk secretion stage (T). Eight *MbWnt genes* were selected for verification by RT-qPCR. The β-actin gene was used as the internal control for the RT-qPCR analysis after screening. As is shown in Fig. 9, six *MbWnt* genes (*MbWnt2*,* MbWnt4*,* MbWnt5a*,* MbWnt7a*,* MbWnt7b*,* MbWnt16*) were significantly highly expressed in vitro culture of musk gland cell at musk secretion stage. In contrary, there was no differential expression of the two *MbWnt* genes (*MbWnt1*,* MbWnt1s*) in vitro culture of musk gland cell at musk secretion stage and non-secretory stage.

## Discussion

The *Wnt* gene is generally believed to have originated before cnidarians, and all existing subfamilies already existed in bilateral animals. Subsequently, it was lost in the branches of platyhelminth and ecdysozoa, and extensive replication occurred in vertebrates. Gene replication is of great significance for generating new genes, and tandem repeats and segmental repeats are important ways for gene replication and expansion [20]. Within multicellular eukaryotic organisms, the *Wnt* gene family is divided into 13 distinct subfamilies. Both humans and mice possess 18 *Wnt* family members, fruit rope has 7 members, and catfish has 20 members. At present, the number of *Wnt* genes in fish genomes is relatively large (20–25), and the reason why the *Wnt* genes in their genomes are rather complex is generally believed to be due to the fact that bony fish have experienced three genome-wide replication events during their evolutionary history [21, 22]. The *MbWnt* gene family has four tandem repeats, but no segmental repeats are found. It is speculated that the *MbWnt* gene family mainly expands through tandem repeats. Despite the remarkable conservation of *Wnt* gene family, members of the *Wnt* gene family have been lost in multiple species [23–25]. In *B. taurus*, the *Wnt* gene family is divided into 12 subfamilies, lacking *WntA*, which is consistent with previous studies [24, 26]. In *B. taurus*, *Wnt9b* and *Wnt3* exhibit tandem repeats on chromosome 19, while *B. indicus* lacks *Wnt9b* on chromosome 19, *Wnt7b* is missing in *Bison bison*, while *B. gaurus* shows losses of both *Wnt9B* and *Wnt16* [27]. *Wnt9* subfamily is unique to symmetrical animals, which was not found in *Chlamys farreri* [28]. This deficiency may be due to the lack of tandem repeat events or gene loss after species undergo tandem repeats during evolution. In the process of genome evolution, gene replication events can lead to the expansion or loss of gene families [29]. The reasons, processes, and mechanisms of gene loss are not yet clear and require further in-depth research. In *B. taurus* and hybrid *B. taurus*, the arrangement order of *Wnt6*,* Wnt10A*, and *Wnt4* on chromosome 2, *Wnt2* and *Wnt16* on chromosome 4, and *Wnt3A*,* Wnt9A*, and *Wnt8A* on chromosome 7 are reversed [27]. The position of *Wnt2B* has changed in *B. taurus* and *B. mutus* [15], which may be due to changes in gene arrangement caused by chromosomal translocation and rearrangement during species evolution [30, 31], or it may be caused by recombination after chromosomal segment inversion [15].

The core motifs and structural domains of gene families are closely related to their functions and activities [32]. The same gene family usually encodes similar motifs and coordinates the regulation of downstream genes [33]. The *Wnt* gene family usually encodes secretory glycoproteins. The *MbWnt* gene is relatively conserved at the 3’end. Except for *MbWnt2s* and *MbWnt9b*, the other members all contain motifs 1, 2, 3, 4, 5, 6, and 8, which may be related to the N-glycosylation site of the *Wnt* protein [34]. In the FMD, All members of *Wnt* gene family have six conserved amino acid sequences, namely motifs 1, 2, 4, 5, 6 and 8, indicating that they have the same functional sites, and these six motifs are very likely to be conserved domains of the *Wnt* protein. It is speculated that these conserved motifs are very likely related to the differentiation of FMD acinar cell. In addition, there are certain differences in the length, quantity, and distribution of introns, UTR, and CDS among the members of the *MbWnt* gene family, which these structural characteristics determine the three-dimensional conformation and function of the *Wnt* protein. Phylogenetic analysis among species is conducive to clarifying the relationship between amino acid sequence similarity and protein functions within the same gene family, which provide a theoretical basis for revealing the functions of genes. In this study, phylogenetic tree analysis revealed that the *Wnt* gene compositions of FMD, *B. taurus*, *Ca. hircus*, and *Ce. elaphus* are similar, all having 12 subfamilies and all lacking the *WntA* family. The deletion of the *Wnt A* gene may be due to the limitations of genomic assembly that it was not expressed, or it was lost during evolution. The specific reasons remain to be further studied.

The *Wnt* gene family is widely present in animals, plants, and microorganisms, participating in various physiological processes and playing an important role in regulating cellular homeostasis [35–38]. It is reported that *Wnt2*,* Wnt3*, and *Wnt5* in zebrafish are widely involved in the processes of neurogenesis, body segment formation and organogenesis [39, 40]. In zebrafish, *Wnt11* is involved in the migration and morphogenesis of early gastrulation and endoderm cells [41], and *Wnt3a* and *Wnt8* combined with oligonucleotides to inhibit the forward of the neuroectoderm, the expansion of the dorsal tissue and the loss of the posterior body structure [41]. In our study, *MbWnt5a* was significantly upregulated in cultured musk gland cells during the musk secretion stage, this may be related to the above functions.

In mouse embryos, *Wnt9b* has been confirmed to be involved in the development of the urogenital system, which serves as a tissue signal for the development of the mesonephritis, hindkidney, and Mullerian duct [42]. The dorsal and ventral axis polarity of amphibians is determined by *Wnt11* and *Wnt5a*, in which is regulated by Dkk-1. The dorsal and ventral axis polarity of zebrafish is determined by Wnt8, in which is inhibited by Sfrp1a and Frzb [43]. It has been reported that *Wnt2*,* Wnt3*,* Wnt4*,* Wnt5*, *Wnt6*,* Wnt7*,* Wnt8*,* Wnt9*, and *Wnt11* are very likely to be directly or indirectly involved in the heart development process of mice and chickens [44]. The relationship between Wnt gene and apoptosis has been widely reported in embryonic development and cell injury. *Wnt1* can inhibit apoptosis through the β-catenin/TCF pathway, and overexpressed *Wnt1* can alleviate cell apoptosis induced by c-myc [45, 46]. Overexpressed *Wnt1* can produce an elongated abdominal foregut in mice, while misexpressed *Wnt1* in gastric epithelial cells can cause the gastric epidermis to shift backward into the duodenum [47]. In fruit flies, the conduction of *Wnt1* signaling is believed to promote the proliferation of small intestinal stem cells and thus is a necessary condition for the midgut regeneration process in fruit flies [48, 49]. Transient expression of *Wnt8* was detected in the hindgut of amphioxus and clawed frogs, while it is believed to be related to the development of the central nervous system in higher vertebrates [50]. *Wnt6* and *Wnt9b* are highly expressed in the intestinal crypt epithelial cells of mice, suggesting that they may have proliferation-driven functions on the epithelial progenitor cells above Paneth cell [51]. In addition, in fruit fly embryos (Stage 13), only weak expression levels of *Wnt6* were detected in the foregut and midgut [52]. In the embryos of mice, clawed frogs and chickens, *Wnt6* tends to be expressed in the ectoderm tissues, which participated in the formation of body segments and the development of limb buds. In adults of mice, *Wnt6* is expressed in the epithelium of various tissues and organs, participating in maintaining the homeostasis of cells [53]. Research has shown that members of the *Wnt* family play a role in ovarian development and hormone secretion, and are involved in various signaling pathways in living organisms. The *Wnt* gene family can enhance the effect of follicle stimulating hormone by activating β - catenin. Both *Wnt4* and *Wnt6* can participate in the selective regulation of chicken follicles. *Wnt4* affects the proliferation of chicken granulosa cells and the synthesis of steroids [54], while *Wnt6* enhances the sensitivity of chicken follicle stimulating hormone receptors to follicle stimulating hormone [55]; *Wnt2*,* Wnt3*,* Wnt5a*, and *Wnt11* play important roles in the pathogenesis of rectal cancer and are expected to become therapeutic targets for rectal cancer [56]. In our study, *MbWnt4* was significantly upregulated in musk gland cells during the musk secretion stage, which regulated the synthesis of steroids, this was consistent with previous research [54].*Wnt10b* in humans and mice plays a regulatory role in the immune system, breast, fat and bones. Abnormal expression of *Wnt10b* can lead to diseases such as breast cancer, hypertrophy and osteoporosis. *Wnt1* is involved in the development of the nervous system and tumorigenesis [56, 57]. Therefore, *Wnt* gene is also an effective candidate gene for disease treatment [57].

## Conclusions

A total of 18 FMD *Wnt* gene family members were identified in this study, which distributed across 8 chromosomes. *MbWnt1*, *MbWnt2*,* MbWnt4*,* MbWnt6*, and *MbWnt9b* contained signal peptides. *MbWnt* proteins shared six conserved motifs. Genome collinearity analysis demonstrated substantial chromosomal correspondence and orthologous conservation between the FMD and the three species of Bovinae and Ce. elaphus. The level of chromosome homology is relatively low, but the level of genome homology is relatively high among these species. We analyzed RNA-seq data of *MbWnt* genes during the non-secretory stage and musk secretion stage. One *MbWnt* gene (*MbWnt7b*) exhibited stage-specific high expression during the musk secretion stage, while two *MbWnt* genes (*MbWnt4*,* MbWnt5a*) demonstrated stage-specific high expression during the non-secretory stage. Furthermore, RT-qPCR analysis revealed that six *MbWnt* genes were significantly upregulated in cultured musk gland cells during the musk secretion stage. Therefore, it is necessary to further study the specific biological functions of these genes as well as their potential regulatory mechanisms in the non-secretory and musk secretion stage.

## Supplementary Information

Additonal file 1: Supplementary Table S1. Predicted protein secondary structure of the *MbWnt* gene family.

Additonal file 2: Supplementary Table S2. GO enrichment of *MbWnt* genes (P<0.05) in the FMD.

Additonal file 3: Supplementary Table S3. qPCR Results Analysis Using the 2^-ΔΔCt Method for Relative Gene Expression.

## Data Availability

In this study, genome and annotation files for forest musk deer ( *Moschus berezovskii* ) were retrieved from MuskDB (http://muskdb.cn/home), the transcriptome datasets and expression analyses are available in the MuskDB and NCBI SRA under accession numbers SRP423226 and SRP060734, and other data were sourced from the following websites: UniProt website (https://www.uniprot.org/); NCBI website (https://www.ncbi.nlm.nih.gov/); Ensembl website (http://useast.ensembl.org/info/data/ftp/index.html).
